# Oxidative stress in the mitochondrial matrix underlies ischemia/reperfusion-induced mitochondrial instability

**DOI:** 10.1016/j.jbc.2022.102780

**Published:** 2022-12-07

**Authors:** Soroosh Solhjoo, Ting Liu, Agnieszka Sidor, Dong I. Lee, Brian O’Rourke, Charles Steenbergen

**Affiliations:** Johns Hopkins University School of Medicine, Baltimore, Maryland, USA

**Keywords:** inner membrane potential oscillations, reactive oxygen species, GSH redox potential, coverslip-induced ischemia, reperfusion, calcium imaging, neonatal rat ventricular myocytes, cardiac monolayers, optical mapping, reentry arrhythmias, GSH, glutathione, GSH-MEE, GSH monoethyl ester, IR, ischemia and reperfusion, NRVM, neonatal rat ventricular myocyte, RIRR, ROS-induced ROS release, ROS, reactive oxygen species, TMRM, tetramethylrhodamine methyl ester

## Abstract

Ischemia and reperfusion affect multiple elements of cardiomyocyte electrophysiology, especially within the mitochondria. We previously showed that in cardiac monolayers, upon reperfusion after coverslip-induced ischemia, mitochondrial inner membrane potential (ΔΨ) unstably oscillates between polarized and depolarized states, and ΔΨ instability corresponds with arrhythmias. Here, through confocal microscopy of compartment-specific molecular probes, we investigate the mechanisms underlying the postischemic ΔΨ oscillations, focusing on the role of Ca^2+^ and oxidative stress. During reperfusion, transient ΔΨ depolarizations occurred concurrently with periods of increased mitochondrial oxidative stress (5.07 ± 1.71 oscillations/15 min, N = 100). Supplementing the antioxidant system with GSH monoethyl ester suppressed ΔΨ oscillations (1.84 ± 1.07 oscillations/15 min, N = 119, *t* test *p* = 0.027) with 37% of mitochondrial clusters showing no ΔΨ oscillations (*versus* 4% in control, odds ratio = 14.08, Fisher’s exact test *p* < 0.001). We found that limiting the production of reactive oxygen species using cyanide inhibited postischemic ΔΨ oscillations (N = 15, *t* test *p* < 10^−5^). Furthermore, ΔΨ oscillations were not associated with any discernable pattern in cell-wide oxidative stress or with the changes in cytosolic or mitochondrial Ca^2+^. Sustained ΔΨ depolarization followed cytosolic and mitochondrial Ca^2+^ increase and was associated with increased cell-wide oxidative stress. Collectively, these findings suggest that transient bouts of increased mitochondrial oxidative stress underlie postischemic ΔΨ oscillations, regardless of Ca^2+^ dynamics.

Reperfusion of the ischemic heart is crucial to limiting cell damage and initiating potential recovery, but it can also play a role in the injury process and trigger cardiac arrhythmias (1, 2, 3, 4, 5). Ischemia and reperfusion (IR) disturb ionic gradients, gap junctional conductivity, and many other factors in cardiomyocyte electrophysiology. These changes transform the postischemic region and the border zone into a heterogeneous substrate—characterized by increased dispersion of refractoriness, slow conduction velocity, and excitation block—which increases the propensity to arrhythmias that can progress to ventricular fibrillation and sudden cardiac death (6).

A major organelle affected by IR is the mitochondrion, the main transformer of oxygen and nutrients into energy. In monolayer cultures of neonatal rat ventricular myocytes (NRVMs), we have shown that regional mitochondrial dysfunction alone, initiated by local perfusion with a chemical mitochondrial uncoupler, leads to inexcitability, slow conduction, and the occurrence of reentrant arrhythmias, mainly *via* opening sarcolemmal ATP-sensitive K^+^ channels (sarcK_ATP_) (7). Furthermore, in an experimental model of coverslip-induced IR in NRVM monolayers (8, 9), we have shown that mitochondrial inner membrane potential (ΔΨ) (10) depolarizes during ischemia and becomes unstable following reperfusion, compromising the functionality of mitochondria as they oscillate between energized and de-energized states or occasionally become fully depolarized (11). The postischemic ΔΨ oscillations are asynchronized not only among the neighboring cardiomyocytes but also among the mitochondrial clusters throughout each cardiomyocyte. The resulting asynchronous instability of mitochondrial function throughout the monolayer might contribute to the heterogeneity in cardiomyocytes’ energy status and ionic concentrations, which likely underlie the postischemic reentrant arrhythmias that occur in this model. The arrhythmogenic mechanisms involved in coverslip-induced IR are more complex than the effect of the chemical uncoupler alone, as inhibition of sarcK_ATP_ channels (with glibenclamide or glimepiride) does not prevent inexcitability during ischemia or the occurrence of reentry during reperfusion (11). IR-induced ΔΨ instability can alter mitochondrial Ca^2+^ handling, further compromising ATP production and potentially affecting cytosolic calcium ([Ca^2+^]_i_) regulation and consequently disrupting the cardiomyocytes’ electromechanical activity. Alternatively, the ionic imbalance, such as Ca^2+^ overload, might be behind ΔΨ oscillations or loss during reperfusion.

Previously, we showed that synchronized ΔΨ oscillations could be triggered in isolated adult cardiomyocytes *via* metabolic stress in the form of substrate deprivation (12) or increased generation of reactive oxygen species (ROS) after a localized laser flash (13). Inhibition of the electron transport chain upstream or downstream of complex III, using rotenone, myxothiazol, or cyanide, stopped spontaneous cell-wide ΔΨ oscillations triggered by a localized laser flash, in concert with significantly decreased ROS accumulation. These observations, on the one hand, suggested complex III as the major site of ROS production during metabolic oscillations and, on the other hand, supported a causative role for oxidative stress in ΔΨ oscillations (13). Cell-wide synchronized ΔΨ oscillations also occurred in adult cardiomyocytes by directly controlling the GSH redox potential (14) (GSH:GSSG ratio, the key indicator of cytosolic redox status) (15). In our experiments in NRVM monolayers, cytosolic GSH became oxidized during coverslip-induced ischemia and recovered on reperfusion (11). 4′-Chlorodiazepam, a ligand for the mitochondrial benzodiazepine receptor (mBZR), slowed the cytosolic GSH pool’s oxidation during ischemia, accelerated its reduction on reperfusion, and prevented postischemic ΔΨ oscillations (11). However, the effect of IR on the redox status of the GSH pool of the mitochondrial matrix has not been explored, and it is unclear whether there is a causal relationship between the cytosolic or mitochondrial redox status and the postischemic ΔΨ instability.

To elucidate the mechanisms underlying IR-induced mitochondrial ΔΨ oscillations, here we investigate the dynamics of the changes in cytosolic and mitochondrial oxidative stress in monolayer cultures of NRVM subjected to coverslip-induced IR. We also test whether the IR-induced changes in mitochondrial or cytosolic Ca^2+^ levels play a role in postischemic mitochondrial instability.

## Results

### Changes in ΔΨ and [Ca^2+^]_m_ during coverslip-induced ischemia

**Figure 1:**
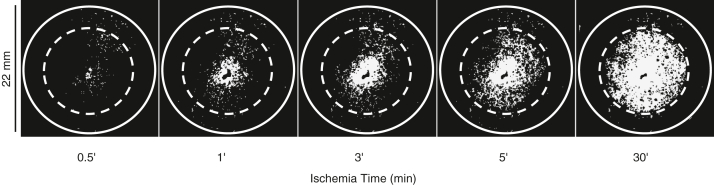
**Coverslip-induced ischemia causes depolarization of mitochondrial inner membrane potential (ΔΨ).** Shortly after the coverslip is placed over the monolayer, a wave of ΔΨ partial depolarization initiates in the center of the ischemic region and propagates toward the edge of the coverslip. Images show the TMRM signal in the dequench mode; the solid line circle shows the edge of the monolayer (22 mm diameter), and the *dashed line* is the edge of the ischemia-inducing coverslip (15 mm diameter).

Shortly after the coverslip was placed on the monolayer, a wave of ΔΨ partial depolarization (11) originated in the center of the ischemic region and slowly spread toward the edge of the coverslip (Fig. 1 and Video S1). By 30 min after the coverslip placement, ΔΨ depolarization had initiated throughout the ischemic region. As previously described, ΔΨ depolarizes during coverslip-induced ischemia in two phases: (1) an initial slow phase that starts upon coverslip placement during which mitochondria partially depolarize, followed by (2) a rapid and more pronounced ΔΨ collapse that starts after up to 30 min after coverslip placement (11). Therefore, ischemia was continued for 60 min so that a uniform area of inexcitable cells that showed contracture and depolarized mitochondria was observed.

**Figure 2:**
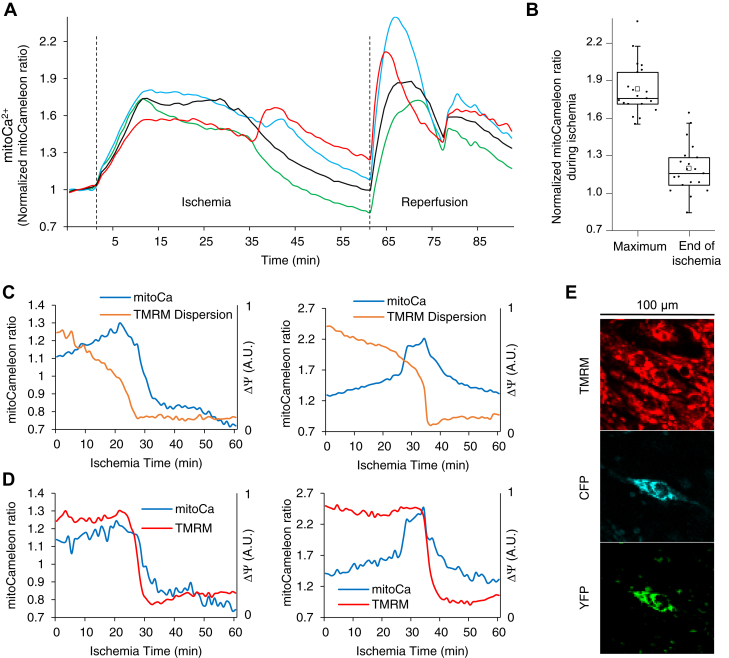
**Dynamics of changes in [Ca**^**2+**^**]**_**m**_**during coverslip-induced IR.***A*, mitoCameleon FRET ratio, normalized to average preischemic values, is shown for four neighboring cells at the center of a monolayer during IR. Cells underwent contracture between 25 to 45 min after the initiation of ischemia. *Dashed lines* show the beginning of ischemia and reperfusion. *B*, the change in mitoCameleon YFP/CFP ratio normalized to the baseline at maximum ratio during and at the end of ischemia (N = 20). *C*, the dynamics of concurrent changes in ΔΨ and [Ca^2+^]_m_ in two cells at the center of the ischemic zone. *D*, the dynamics of changes in ΔΨ (TMRM intensity) and [Ca^2+^]_m_ in individual mitochondrial clusters in each of the cells in (*C*). *E*, the confocal microscopy images, magnified and cropped (100 × 100 μm^2^) around the cell corresponding to the *left panel* in (*C*) at the beginning of the ischemic period. TMRM signal is in linear mode. Not all cells expressed the mitoCameleon Ca^2+^ indicator. IR, ischemia and reperfusion; TMRM, tetramethylrhodamine methyl ester.

[Ca^2+^]_m_ initially increased during ischemia (+82.84 ± 4.61% mitoCameleon FRET ratio change compared to baseline, N = 20) and decreased as mitochondria depolarized and the cells showed contracture (+19.69 ± 4.42% mitoCameleon FRET ratio change compared to baseline, N = 20; Fig. 2). However, the changes in [Ca^2+^]_m_ varied among cardiomyocytes reflecting the various dynamics of ΔΨ and [Ca^2+^]_i_ during IR.

### Postischemic ΔΨ instability and redox status

After the coverslip was lifted, mitochondria became unstable and ΔΨ of individual mitochondrial clusters oscillated throughout the reperfused region (Video S2). We tested whether oxidative stress during IR played a role in postischemic ΔΨ instability.

**Figure 3:**
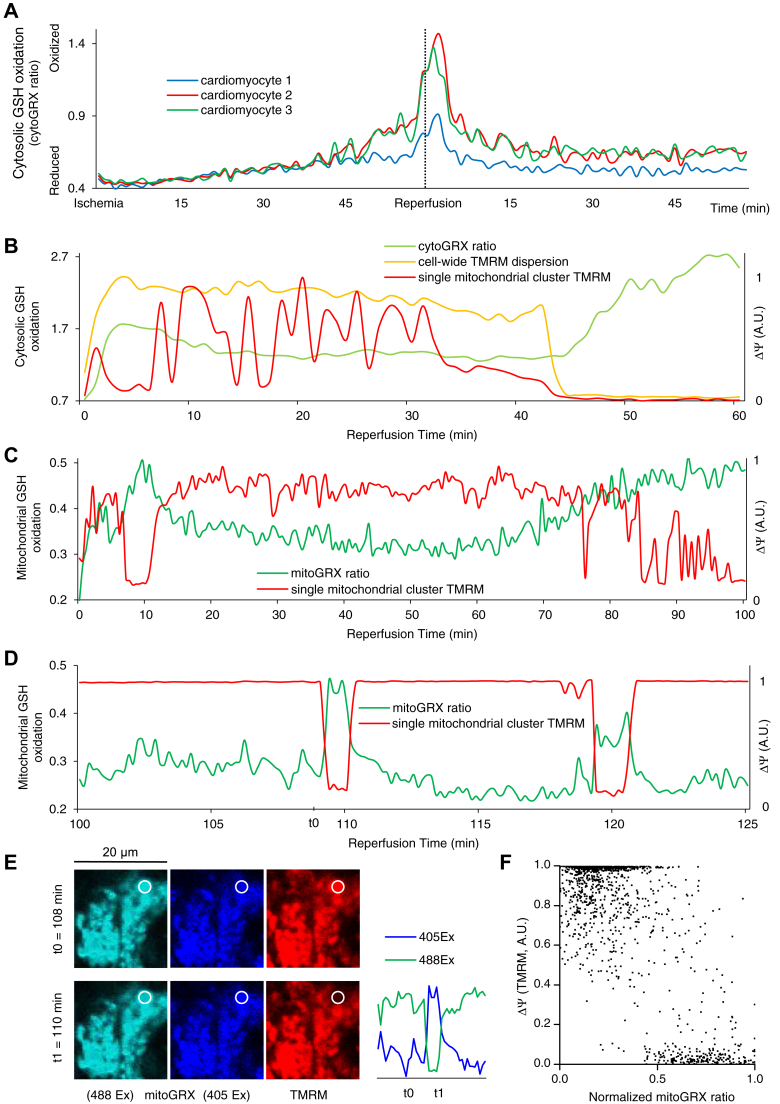
**Oxidative stress plays a significant role in IR-induced ΔΨ instability.***A*, cytosolic GSH oxidized during ischemia. On reperfusion, GSH oxidized further early during reperfusion and then partially recovered. CytoGRX ratio (GSSG:GSH) is shown in three cardiomyocytes in the center of a monolayer during IR. The dashed line shows the beginning of reperfusion 60 min. *B*, transient ΔΨ oscillations during reperfusion do not seem to correlate with the changes in cytosolic glutathione redox potential; however, sustained depolarization of the mitochondrial network of the cardiomyocyte is followed by a further increase in cytosolic oxidative stress. *C* and *D*, postischemic ΔΨ oscillations correspond with mitochondrial GSH redox potential. ΔΨ depolarization correlates with GSH oxidation, and ΔΨ recovery correlates with GSH reduction. *E*, a section of a cell in the center of the reperfused region of a monolayer loaded with TMRM (50 nM) and transfected with mitoGRX. The images correspond with the first oscillation in ΔΨ and mitochondrial redox status in (*E*) at t0 and t1. The emission signals for each excitation wavelength are shown on the *right*. *F*, scatter plot of normalized TMRM measurements *versus* normalized mitoGRX ratios during reperfusion; aggregate data for nine mitochondrial clusters in separate monolayers subjected to IR are shown. IR, ischemia and reperfusion; TMRM, tetramethylrhodamine methyl ester.

During ischemia, cytoGRX F405/F488 ratio (GSSH:GSH) increased, indicating oxidation of cytosolic GSH pool, consistent with activation of the antioxidant pathway in response to accumulation of ROS during hypoxia. On reperfusion, an initial rapid increase in the oxidation of cytosolic GSH was observed, followed by partial recovery (Fig. 3, *A* and *B*). Occasionally, prolonged reperfusion led to sustained loss of ΔΨ, which was associated with further cytosolic GSH oxidation as the cardiomyocytes developed contracture (Fig. 3*B*). CytoGRX ratio increased to 50% of its maximum oxidized level 5.2 ± 1.03 min following 50% loss of cell-wide tetramethylrhodamine methyl ester (TMRM) signal (N = 10 experiments).

There was no apparent connection between ΔΨ oscillations of individual mitochondrial clusters across the cardiomyocyte and the dynamics of cytosolic thiol oxidation. Close examination of the CytoGRX signal revealed no consistent discernable change in the cytosolic GSSG:GSH during ΔΨ oscillations (Fig. 3*B*). In contrast, measurement of the mitochondrial GSSG:GSH with mitoGRX revealed marked bursts of matrix GSH oxidation during individual ΔΨ oscillations (Fig. 3, *C*–*F*). However, with more disorganized ΔΨ oscillations, GSH oxidation in the mitochondrial matrix and ΔΨ depolarization did not have a one-to-one relationship. An extended period of oxidative stress could be concurrent with several cycles of transient ΔΨ depolarization (Fig. 3*C*, during late reperfusion). These findings establish an interdependence between the level of mitochondrial oxidative stress and the occurrence of ΔΨ oscillations.

In our recordings, the mitoGRX ratio increased concurrently with or shortly after ΔΨ depolarization. However, since the mitoGRX signal takes about 20 s to reach a plateau (16), ΔΨ depolarizations might, in fact, be following the increases in oxidative stress in the matrix. Therefore, we tested whether limiting mitochondrial oxidative stress could inhibit ΔΨ depolarizations.

**Figure 4:**
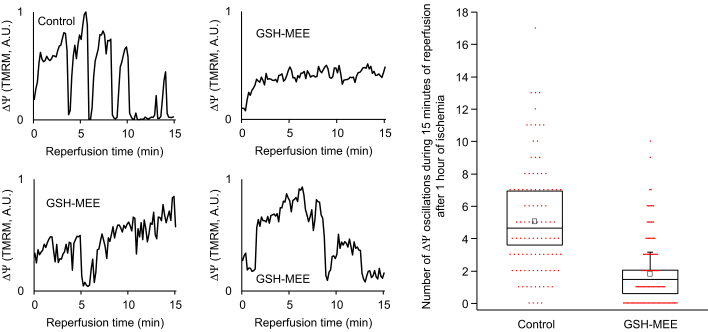
**Boosting the antioxidant pool suppresses IR-induced ΔΨ instability.***Left:* Example recordings of ΔΨ (TMRM intensity in individual mitochondrial clusters) during reperfusion after 1 h of ischemia in control and monolayers treated with GSH-MEE. *Right:* Average numbers of transient ΔΨ depolarizations in the first 15 min of reperfusion after 1 h ischemia in control monolayers (5.07 ± 1.71 oscillations/15 min, N = 3) and monolayers preincubated with GSH-MEE (1.84 ± 1.07 oscillations/15 min, N = 4; *t* test *p* = 0.027) are shown. For each monolayer, the number of ΔΨ oscillations is averaged over 20 to 40 randomly selected mitochondrial clusters in a 150 × 150 μm^2^ area in the center of the reperfused zone. Aggregate individual datapoints, including 100 individual mitochondrial clusters for control and 119 individual mitochondrial clusters with GSH-MEE treatment, are shown with *red* dots. GSH-MEE, GSH monoethyl ester; IR, ischemia and reperfusion; TMRM, tetramethylrhodamine methyl ester.

Supplementing the Tyrode’s solution with GSH monoethyl ester (GSH-MEE, a lipid membrane–permeable GSH derivative, 5 mM, 3 h incubation before and during IR) significantly lowered the frequency of postischemic ΔΨ oscillations (1.84 ± 1.07 oscillations/15 min, compared to 5.07 ± 1.71 oscillations/15 min in control; *p* = 0.027). In the presence of GSH-MEE, 37% of the analyzed mitochondrial clusters showed no ΔΨ oscillations during the first 15 min of reperfusion, compared to only 4% in control (odds ratio = 14.08, Fisher’s exact test *p* < 0.001, N = 119 for GSH-MEE, N = 100 for control, Fig. 4).

**Figure 5:**
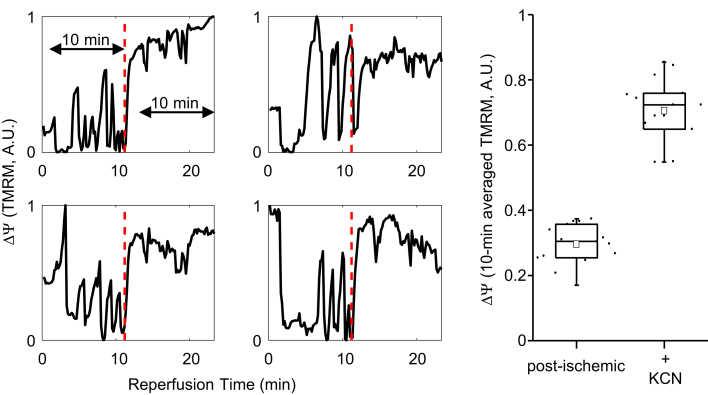
**Cyanide inhibits postischemic ΔΨ instability.***Left:* Changes in ΔΨ (normalized TMRM intensity) are shown for four distinct mitochondrial clusters during reperfusion after 1 h of coverslip-induced ischemia. Adding KCN to the media during reperfusion (minute 11, *vertical red dashed line*) eliminated ΔΨ oscillations. *Right:* The 10 min averaged ΔΨ becomes significantly larger after adding KCN (N =15, *t* test *p* < 10^−5^). The 10 min period, as shown in the first diagram on the left, started 2 min after the onset of KCN perfusion. TMRM, tetramethylrhodamine methyl ester.

We also tested whether cyanide could stabilize ΔΨ during reperfusion. Cyanide works by arresting electron flow at complex IV, resulting in the reduction of the FeS(III) electron acceptor of complex III. The lack of an oxidized acceptor shuts down the Q-cycle and inhibits ROS production at complex III (17), a major site of ROS production in the postischemic heart (18, 19, 20). Adding cyanide (KCN, 2 mM) to the reperfusate inhibited ΔΨ oscillations (Fig. 5; TMRM_baseline_ = 0.3 ± 0.02, TMRM_KCN_ = 0.7 ± 0.03, averaged normalized TMRM signal recorded for 10 min at baseline and for 10 min starting 2 min after adding KCN, N = 15, *p* < 10^−5^).

### Postischemic ΔΨ instability and Ca^2+^

**Figure 6:**
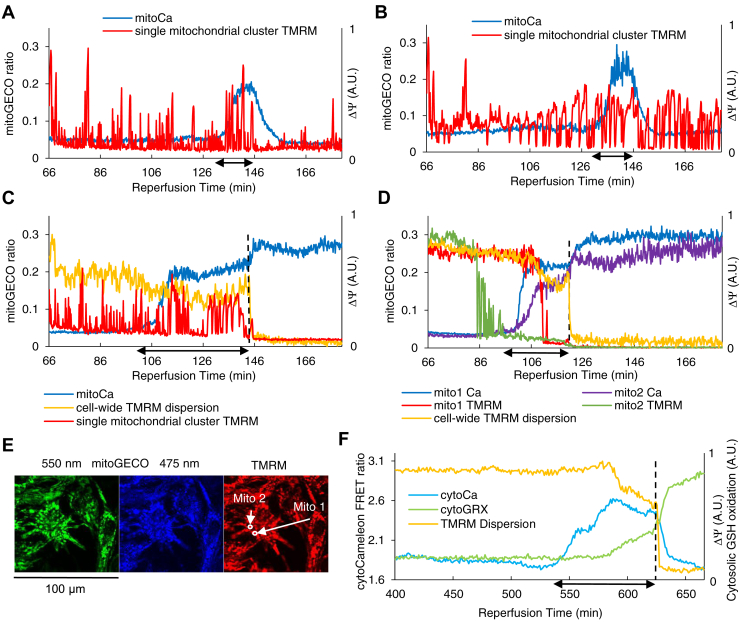
**ΔΨ oscillations are independent of the changes in [Ca**^**2+**^**]**_**m**_**.***A* and *B*, ΔΨ oscillations occur during reperfusion irrespective of the changes in [Ca^2+^]_m_ in two neighboring mitochondrial clusters in a single cell in the center of an NRVM monolayer. [Ca^2+^]_m_ increases most likely due to [Ca^2+^]_i_ increase as the cell twitches during late reperfusion and decreases after the twitching stops. *C* and *D*, in two separate cardiac myocytes in the center of an NRVM monolayer, [Ca^2+^]_m_ increases as the reperfused cardiac myocytes start twitching. Single mitochondrial clusters depolarize or oscillate irrespective of the changes in [Ca^2+^]_m_. The membrane potential of the whole mitochondrial network of the cells collapses as they undergo contracture. *E*, fluorescence microscopy images of TMRM and mitoGECO signals specifying the mitochondrial clusters used in panel (*D*). Separate measures of emission wavelengths for (*A*–*D*) are shown in Fig. S1. *F*, [Ca^2+^]_i_ increased prior to sustained mitochondrial depolarization during reperfusion. Concurrent imaging of cytosolic GSH redox potential showed a gradual increase in GSH oxidation prior to sustained ΔΨ depolarization. The numbers on the horizontal axes show the time in minutes since the beginning of reperfusion after an hour of ischemia; in (*A*–*D*), tick marks are placed every 10 min and only the first 2 labels are shown. The dashed lines in (*C*, *D*, and *F*) show the time of sustained depolarization and contracture. Horizontal arrows (↔) along the time axis indicate the periods of spontaneous contractions. NRVM, neonatal rat ventricular myocyte; TMRM, tetramethylrhodamine methyl ester.

During reperfusion, there was a transient increase in [Ca^2+^]_m_, followed by recovery in cardiomyocytes with a period of spontaneous contractions followed by quiescence (Fig. 6, *A* and *B* and Video S3). The spontaneous contractions and the changes in [Ca^2+^]_m_ were not coupled with neighboring cardiomyocytes. Prior to the incidence of sustained ΔΨ depolarization throughout the cell and contracture, the cardiomyocytes showed spontaneous contractions, and [Ca^2+^]_m_ and [Ca^2+^]_i_ increased (Fig. 6, *C*–*F* and Video S3). Spontaneous activity leading to increased [Ca^2+^]_m_ and [Ca^2+^]_i_ and sustained ΔΨ depolarization events occurred infrequently and mainly during long reperfusion periods; therefore, we have chosen specific periods of experiments with prolonged reperfusion to show these phenomena in Figure 6.

These significant changes in [Ca^2+^]_m_ did not seem to be associated with any specific patterns in ΔΨ oscillations: individual mitochondrial ΔΨ continued oscillations (Fig. 6*C*) or reached a near-depolarization level after a few oscillations (Fig. 6*D*), while [Ca^2+^]_m_ increased. ΔΨ fully depolarized upon the collapse of the whole mitochondrial network of the cardiomyocyte.

**Figure 7:**
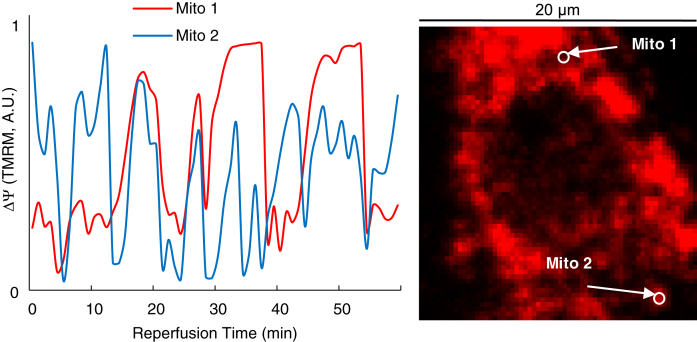
**ΔΨ oscillations occur in Ca**^**2+**^**-depleted conditions.** ΔΨ oscillations in two randomly selected mitochondrial clusters in a cardiomyocyte in the reperfused region of a monolayer after 1 h ischemia in Ca^2+^-free Tyrode’s solution in the presence of EGTA and ionomycin. The monolayer was preincubated with EGTA, thapsigargin, and caffeine. ΔΨ oscillations occurred in almost all the cardiac myocytes in the field of view in two separate experiments.

ΔΨ oscillations also occurred when IR was induced in Ca^2+^-depleted conditions in the presence of EGTA (5 mM) and ionomycin (6 μM) after incubation with EGTA (5 mM), thapsigargin (1 mM), and caffeine (10 mM) (Fig. 7).

## Discussion

In the present work, we investigate the mechanisms involved in IR-induced mitochondrial instability; specifically, we show the role of oxidative stress in mitochondrial dysfunction during reperfusion after coverslip-induced ischemia in cardiac monolayers. Our key findings include (1) postischemic ΔΨ oscillations are concurrent with transient increases in mitochondrial oxidative stress during reperfusion; (2) limiting the level of oxidative stress could inhibit ΔΨ oscillations; (3) sustained ΔΨ depolarization is associated with an increase in cytosolic GSH oxidation; (4) sustained ΔΨ depolarizations follow mitochondrial Ca^2+^ overload but ΔΨ oscillations are independent of mitochondrial Ca^2+^.

Determining the cellular and subcellular mechanisms involved in IR has been a challenge, which the development of new models of IR and novel molecular biology techniques can help overcome. A significant milestone in resolving the molecular mechanisms involved in IR was the introduction of the coverslip-induced IR model in confluent monolayers of cultured cardiomyocytes (8). In this two-dimensional model, the limited supply of oxygen and nutrients, the entrapment of metabolites, and the decrease in pH (Fig. S2) under the coverslip closely mimic the pathophysiological conditions of ischemia. In contrast, other models of IR, such as hypoxia, anoxia, or metabolic inhibition, only simulate a small fraction of the ischemic conditions. The coverslip-induced ischemia is rapidly reversible, simulating reperfusion. This platform has also been used to reproduce and investigate IR-induced arrhythmic events (11, 21). Using this model, we previously showed the biphasic depolarization of the mitochondrial inner membrane during ischemia. Utilization of the limited supply of glucose and glycolytic reserves along with ATP synthase reversal might underlie the delay in ΔΨ collapse during ischemia. We also showed the unstable ΔΨ recovery during reperfusion that manifested as ΔΨ oscillations and correlated with the occurrence of wavelets and reentry (11).

Our present work reveals that IR-induced ΔΨ oscillations are associated with temporary increases in the level of oxidative stress in the matrix of individual mitochondrial clusters as measured by the GSSG:GSH ratio. A transient increase in oxidative stress in the matrix correlated with a transient ΔΨ depolarization, while a more extended period of increased oxidative stress could be accompanied by multiple cycles of ΔΨ oscillations (Fig. 3, *C*–*E*). Due to the high spatial and temporal heterogeneity of the postischemic ΔΨ oscillations in NRVM monolayers, whole-tissue analysis methods such as mass spectrometry are not appropriate to study these oscillations; hence, our localized temporal correlation approach.

Our group previously showed that in individual isolated adult guinea pig ventricular myocytes, after a localized laser flash increased the cytosolic ROS above a critical threshold, self-sustaining synchronized cell-wide ΔΨ oscillations occurred and correlated with oscillations in cytosolic ROS level (22, 23). Similar events were observed through controlled perturbations in the GSSG:GSH ratio (14). These observations suggested ROS-induced ROS release (RIRR) as the mechanism behind ΔΨ oscillations and that a critical level of oxidative stress was needed to trigger ΔΨ oscillations (22, 24).

ΔΨ instability in postischemic NRVM monolayers appears to be somewhat different from that of stressed adult cardiomyocyte models. Contrary to the synchronized ΔΨ oscillations of the whole mitochondrial network seen in the adult cardiomyocyte, during reperfusion of ischemic NRVM monolayers, individual mitochondrial clusters oscillate asynchronously with respect to other mitochondria within each cardiomyocyte, and as seen here, the cytosolic GSSG:GSH ratio does not oscillate in the postischemic monolayer during these random ΔΨ oscillations.

RIRR involves the activation of energy-dissipating mitochondrial ion channels (25, 26), posited to involve the mitochondrial permeability transition pore (mPTP) (27) or the inner membrane anion channel (IMAC), an anion channel putatively regulated by mBZR (28, 29, 30). We previously showed that in NRVM monolayers subjected to coverslip-induced IR, supplementing the perfusate with 4′-chlorodiazepam (a ligand for mBZR) prevented ΔΨ oscillations and stabilized mitochondrial recovery in the energized state (11). Cyclosporin A (an mPTP inhibitor) did not prevent postischemic ΔΨ oscillations. However, late sustained ΔΨ depolarization was less frequently observed with cyclosporin A (11). In a model of IR in cultured adult rat cardiomyocytes, where ischemia was simulated by 3 h of anoxia and acidosis and reperfusion by reoxygenation and pH recovery, Kim *et al*. (31) reported observing ΔΨ oscillations during reperfusion (data were not shown), which ended in sustained ΔΨ depolarization and cell death. Similar to our prior findings (11), they reported that in the presence of cyclosporin A, mitochondria did not undergo sustained depolarization, Ca^2+^ did not overload mitochondria or cytosol, and cell death was prevented (31). Their experiments suggested oxidative stress and pH recovery as the causes and Ca^2+^ overload as a consequence of mPTP opening during reperfusion. However, in our experiments, [Ca^2+^]_m_ and [Ca^2+^]_i_ increase before sustained ΔΨ depolarizations, implicating the activation of the classical Ca^2+^-dependent mPTP. Our findings indicate that in the coverslip-induced IR model, early postischemic ΔΨ oscillations involve transient openings of a Ca^2+^-independent ROS-activated channel (postulated to be IMAC), followed by sustained ΔΨ depolarization mediated by mPTP.

Our present findings support the hypothesis that during each cycle of IR-induced ΔΨ oscillation, the accumulation of ROS in the matrix over a critical threshold triggers IMAC opening and ΔΨ depolarization. ROS is released from the mitochondrial matrix through IMAC and scavenged by the antioxidant system, while ROS production is decreased during the mitochondrial uncoupling. The resulting decrease in ROS levels permits the closing of the energy-dissipating channel and the rebuilding of ΔΨ. Sustained oscillation and prolongation of the depolarized periods will eventually exhaust the antioxidant system of the cell and contribute to further, more dramatic, oxidation of cytosolic and mitochondrial GSH pools, causing additional dysfunction of the subsystems of the cell, resulting in uncontrollable cytosolic and mitochondrial Ca^2+^ overload. The gradual increase in cytosolic oxidative stress seen in some experiments before sustained ΔΨ depolarization (Fig. 6*F*) suggests that a critical level of oxidative stress can contribute to the sustained ΔΨ depolarization associated with Ca^2+^ overload.

Supplementing the GSH pool of the cardiomyocytes with GSH-MEE lowered the frequency of ΔΨ oscillations during reperfusion. In intact isolated hepatocytes, ROS increased due to exposure (≥ 30 min) to a mixture of iodoacetate and cyanide (32). In intact cardiomyocytes subjected to oxidative stress or IR, exposure to cyanide, at least in the short term, inhibits mitochondrial electron transport and, therefore, ROS production by complex III (13, 33). This prevents ROS accumulation in cardiomyocytes and suppresses RIRR. In line with our previous findings on the effect of cyanide on the metabolic oscillations in adult cardiomyocytes (13), ΔΨ oscillations were diminished by acute treatment with KCN during reperfusion after coverslip-induced ischemia. These observations also conform with the findings in whole rat hearts subjected to a short IR protocol (5 min of ischemia followed by 5 min of reperfusion), where including cyanide in the reperfusate diminished ROS generation and improved the recovery of myocardial contractility (33). These findings suggest that restricting oxidative stress might be a strategy to protect mitochondrial function in the postischemic myocardium.

**Figure 8:**
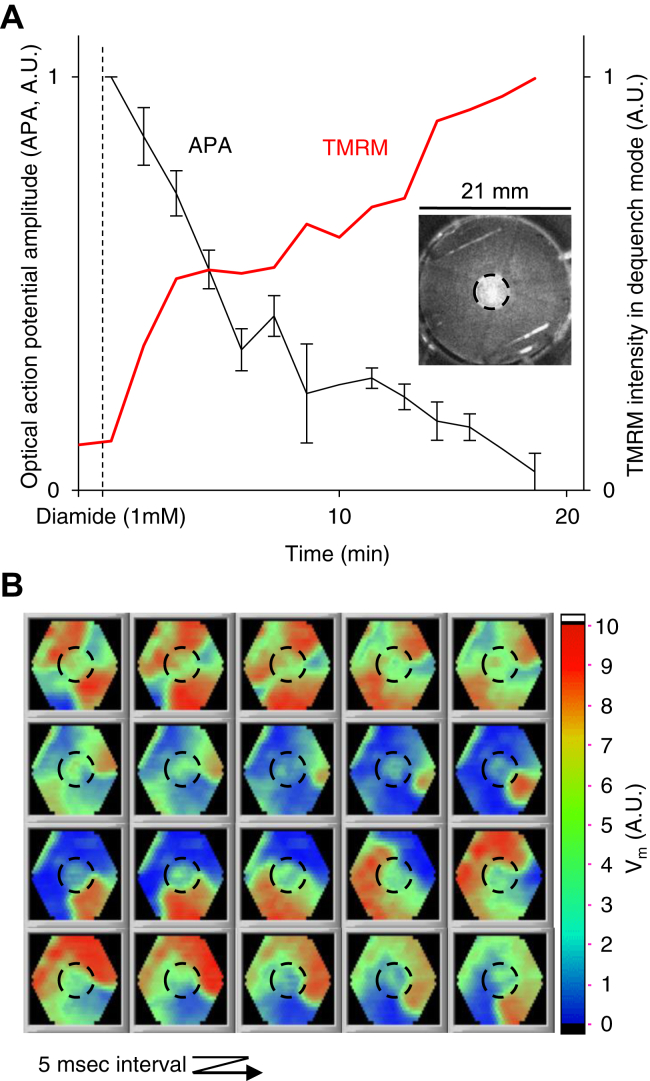
**Oxidative stress depolarizes the mitochondria and can be a significant arrhythmogenic factor.***A*, local perfusion of the center (D = 5 mm) of NRVM monolayers with a thiol oxidizing agent (diamide, 1 mM) causes ΔΨ depolarization and inexcitability (in separate experiments). Error bars for action potential amplitude (APA) show the SEM (N = 4). *Inset:* A monolayer 20 min after local perfusion with diamide; mitochondria are loaded with TMRM at 2 μM in dequenching mode; the bright region indicates depolarized mitochondria. *B*, optical mapping of V_m_ shows the initiation and maintenance of a reentrant wave. Reentry occurred in 8 out of 10 monolayers perfused with diamide *versus* none in control monolayers (*p* < 0.001). A change from *blue* to *red* indicates the depolarization of the cell membrane. NRVM, neonatal rat ventricular myocyte; TMRM, tetramethylrhodamine methyl ester.

In postischemic NRVM monolayers, ΔΨ instability is associated with arrhythmias (11), and mitochondrial dysfunction might be a key factor underlying postischemic arrhythmias. Using computational and experimental models, we previously showed the arrhythmogenic effects of mitochondrial dysfunction (7). When we oxidized the cellular GSH pool, mitochondria depolarized, action potential amplitude diminished, and reentry occurred in 8 out of 10 monolayers (compared to none in untreated monolayers, Fisher’s exact test: *p* < 0.001, Fig. 8). IR-induced oxidative stress could destabilize mitochondrial function and, in part, contribute to arrhythmogenesis as a myriad of IR-induced changes in ion balance and energy level can render the substrate prone to triggered automaticity and reentry. ΔΨ instability can compromise cytosolic and mitochondrial Ca^2+^ handling and lower the resources for ATP generation. Bouts of mitochondrial depolarization compromise oxidative phosphorylation, and the depolarized mitochondria can further consume ATP. The resulting increase in ADP/ATP ratio can contribute to the myocardium's ion imbalance and electrophysiological heterogeneity. While supplementing the GSH pool of cardiac myocytes inhibited postischemic ΔΨ instability, reperfusion reentry could still occur in monolayers treated with GSH-MEE (Videos S4–S6), attesting to the multifactorial nature of IR arrhythmias.

## Experimental procedures

### Monolayer cultures of neonatal rat ventricular myocytes

NRVMs were isolated from 2-day-old Sprague–Dawley rats (Harlan Laboratories) as described previously (11). All experimental procedures were approved by the Institutional Animal Care and Use Committee of Johns Hopkins University. One million NRVMs were cultured on each fibronectin-coated circular cover glass (D = 22 mm) and superfused with a medium containing 10% fetal bovine serum. The medium was changed every day. After 4 to 5 days of culture, confluent monolayers were used for IR experiments.

### Fluorescent dye and molecular probes

To measure oxidative stress, pH-insensitive ratiometric molecular probes for measuring cytosolic or mitochondrial GSSG:GSH redox levels, cyto-Grx1-roGFP2 (cytoGRX) or mito-Grx1-roGFP2 (16) (mitoGRX), were transduced into the NRVMs using adenoviral gene transfer vectors (34) on the second day of culture (excitation: 405 nm and 488 nm, emission: 525 nm; GSSG:GSH ↔ F405/F488 ratio). For measuring cytosolic Ca^2+^ levels ([Ca^2+^]_i_), we developed an adenovirus to express the FRET-based Ca^2+^ probe, Cameleon (CFP/YFP), in the cytosol (cytoCameleon). Mitochondria-targeted Cameleon (35) (mitoCameleon) was used for measuring real-time mitochondrial Ca^2+^ levels ([Ca^2+^]_m_) during ischemia. To measure [Ca^2+^]_m_ during reperfusion, we used the ratiometric genetically encoded probe (36) mito-GEM-GECO (mitoGECO; excitation: 405 nm, emission: 475 nm: Ca^2+^-bound, 550 nm: Ca^2+^-free). The cells were loaded with the potentiometric fluorescent dye, TMRM (50 nM; excitation: 561 nm, emission: 617 nm) for 1 h before the IR experiment when recording ΔΨ at high magnification using confocal microscopy (FV3000, Olympus, Inc). Dispersion of the TMRM signal throughout a cardiomyocyte was calculated and used to measure cell-wide ΔΨ, as described before (11). For imaging ΔΨ across the whole monolayer, we used the Olympus MVX10 macro fluorescence imaging system, and cells were loaded with TMRM at 2 μM for 2 h so that TMRM fluorescence dequenching indicated ΔΨ depolarization (37).

### Ischemia/reperfusion

To induce IR in a monolayer, we placed the monolayer in the chamber of the imaging system and perfused it with Tyrode’s solution consisting of (in mmol/l) the following: 135 NaCl, 5.4 KCl, 1.8 CaCl_2_, 1 MgCl_2_, 0.33 NaH_2_PO_4_, 5 Hepes, and 5 glucose at 37 °C. The monolayer was electrically paced at 1 Hz. Ischemia was induced by placing a glass coverslip (D = 15 mm, #1, Fisher Scientific) on the center of the monolayer. This system restricts cellular access to oxygen and nutrients while metabolites accumulate in the limited extracellular space (8, 9, 11, 21). Substrates and oxygen are restored by removing the cover glass after 1 h of ischemia to simulate reperfusion. As shown previously, if the myocytes are cultured on a semipermeable membrane before applying the coverslip, maintained oxygen and metabolite exchange prevents any significant changes in APD, calcium transient duration, or conduction velocity (21). Therefore, the observed physiological changes are not due to the mechanical effects of cover glass placement (8, 21). Images were typically recorded every 1 min during ischemia and every 10 s during reperfusion.

### Optical mapping of the sarcolemmal membrane voltage

The changes in sarcolemmal membrane potential were mapped with a 464-photodiode array using the voltage-sensitive fluorescent dye, di-4-ANEPPS (5 μM), as the voltage waves, elicited by a stimulation pulse train (1 Hz), propagated through the monolayer. The data acquisition, processing, and visualization for the optical mapping setup were done using custom-made software written in LabVIEW (National Instruments, Inc).

### Regional GSH oxidation

Monolayers were cultured on fibronectin-coated plastic coverslips (D = 21 mm) for 5 to 7 days. Using a previously described custom-built apparatus (7), a thiol oxidizing agent (diamide, 1 mM) was locally perfused in the center part (D = 5 mm) of the monolayer, while the rest of the coverslip received normal Tyrode's solution.

### Data analysis

ImageJ (https://imagej.net/software/fiji/) (38) was used for image analysis. Microsoft Excel (Microsoft), MATLAB (MathWorks), and Origin (OriginLab) were used for data tabulation, analysis, and presentation. The data are reported as mean ± SD unless mentioned otherwise. Student’s *t* test and Fisher’s exact test were used for statistical analysis, and *p*-values < 0.05 were considered statistically significant.

## Data availability

The data are available from the corresponding author upon reasonable request.

## Supporting information

This article contains supporting information.

## Conflict of interest

The authors declare that they have no conflicts of interest with the contents of this article.
