# Supplementary material for: Oxidative stress in the mitochondrial matrix underlies ischemia/reperfusion-induced mitochondrial instability
Source: J Biol Chem. 2022 Dec 7;299(1):102780. doi: 10.1016/j.jbc.2022.102780 (PMC9852550; doi:10.1016/j.jbc.2022.102780)
Supplement: Supplementary material [file mmc7.pdf]

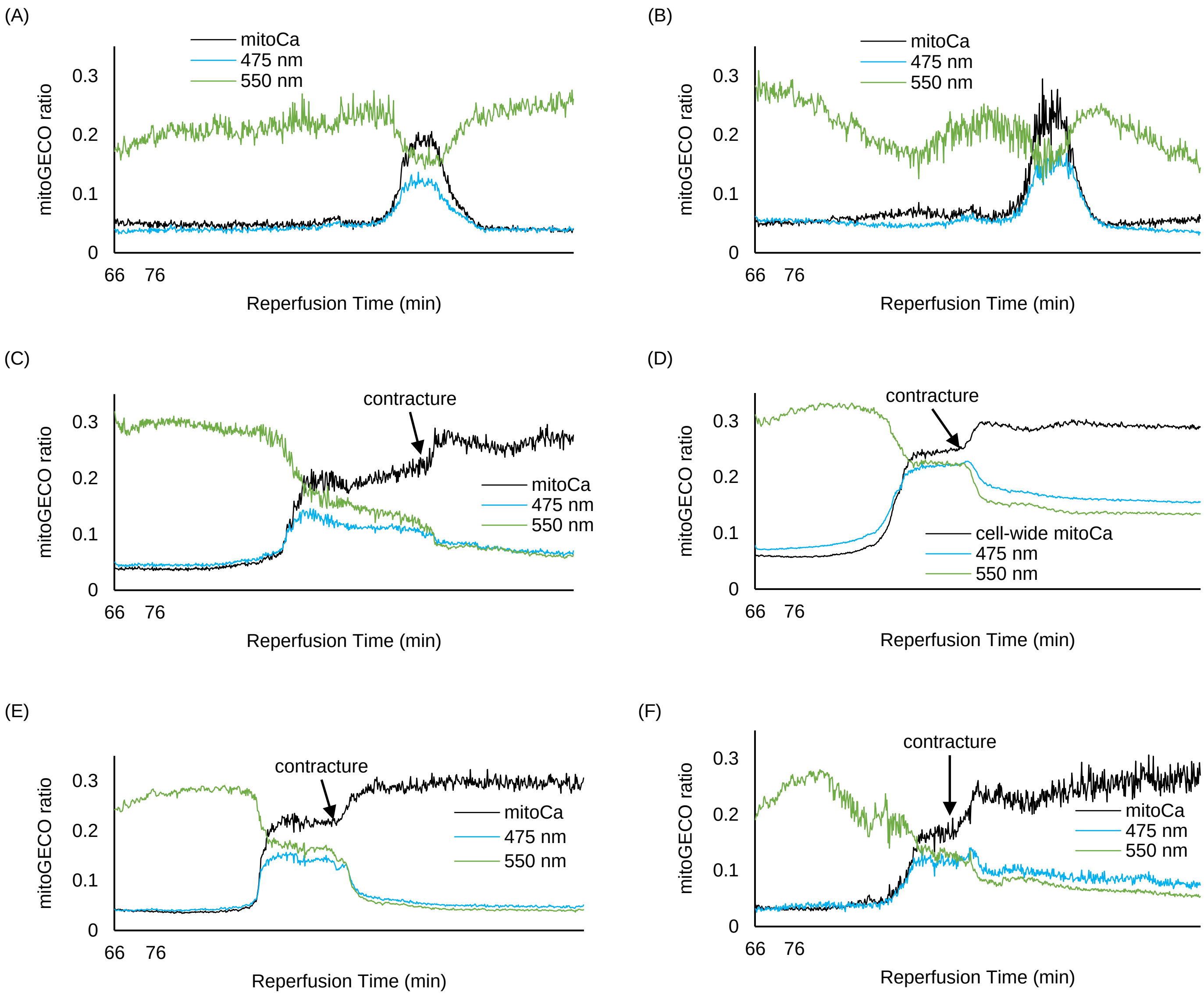

Figure S1. Raw data corresponding to Figure 6. The diagrams show the changes in Ca^{2+}-bound (475 nm) and Ca^{2+}-free (550 nm) emissions during reperfusion, corresponding to Figure 6 panels A to D. **A:** 6A; **B:** 6B; **C:** 6C; **D:** whole-cell measurements for 6D; **E:** Mito 1 mitochondrial cluster in 6D; **F:** Mito 2 mitochondrial cluster in 6D. **C-F:** The decrease in both emission wavelengths after contracture suggests that mitoGECO exits the cell as it undergoes contracture. 475 nm signals have been magnified for clearer presentation.

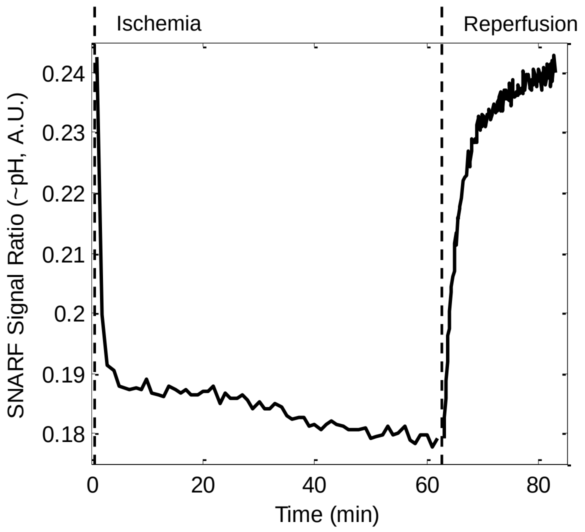

Figure S2. Dynamics of changes in pH during coverslip-induced IR. Early after coverslip placement, pH drops rapidly (to around 5 as estimated by Pitts and Toombs, ref. 8), indicating a switch to glycolysis. Imaging was done by confocal microscopy using pH-sensitive fluorescent dye SNARF.

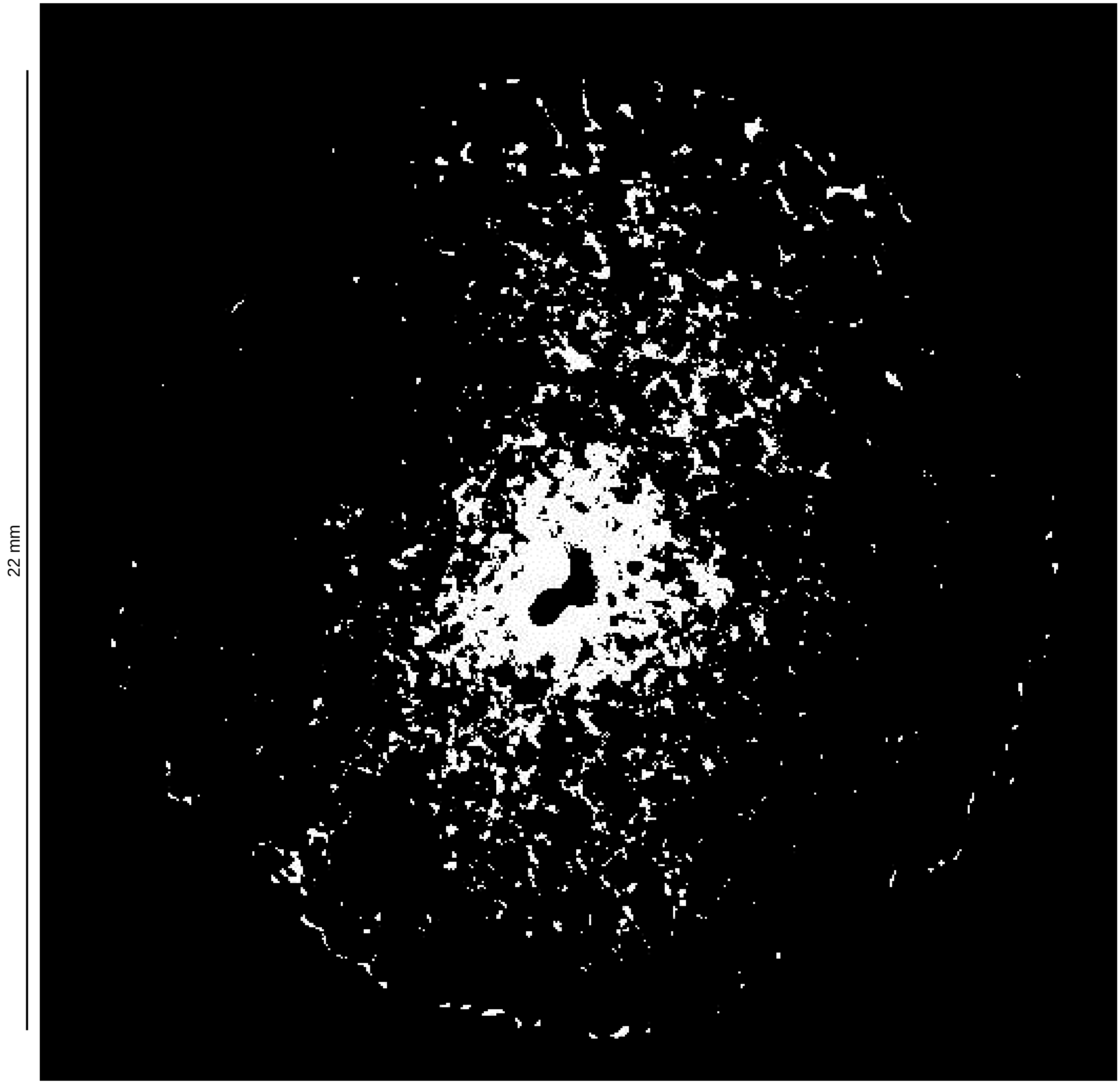

Video S1. Mitochondrial depolarization during coverslip-induced ischemia. Shortly after the coverslip was placed on the monolayer, a wave of $\Delta\Psi$ partial depolarization originated in the center of the ischemic region and slowly spread towards the edge of the coverslip.

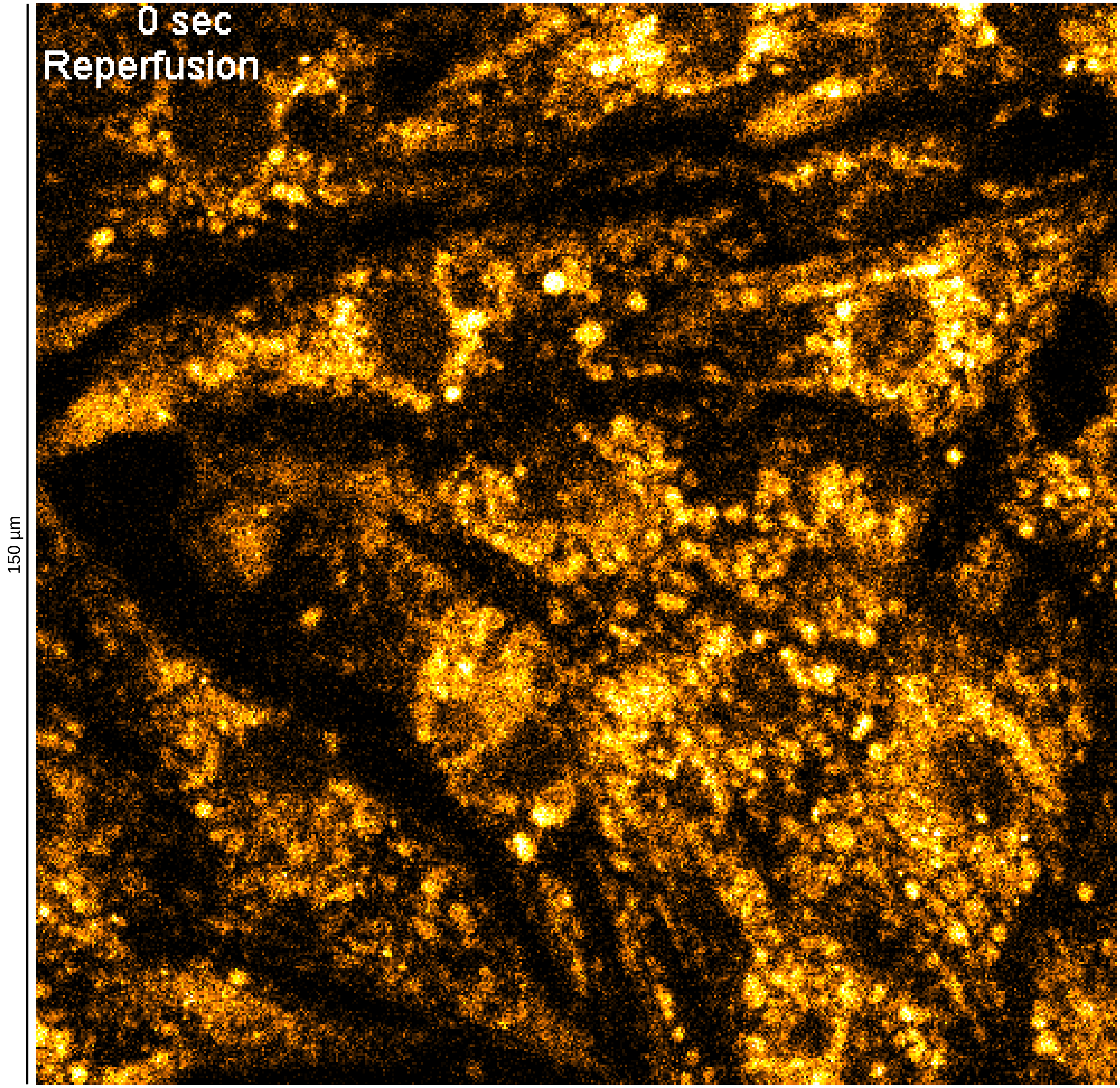

Video S2. Mitochondrial instability during reperfusion after coverslip-induced ischemia. $\Delta\Psi$ oscillations are seen as the flickering of the TMRM signal in the post-ischemic section of the NRVM monolayer. Video shows 1 hour of confocal imaging of 150x150 µm^2 area in the center of a TMRM-loaded monolayer during reperfusion after 1 hour of ischemia; time resolution: 30 sec.

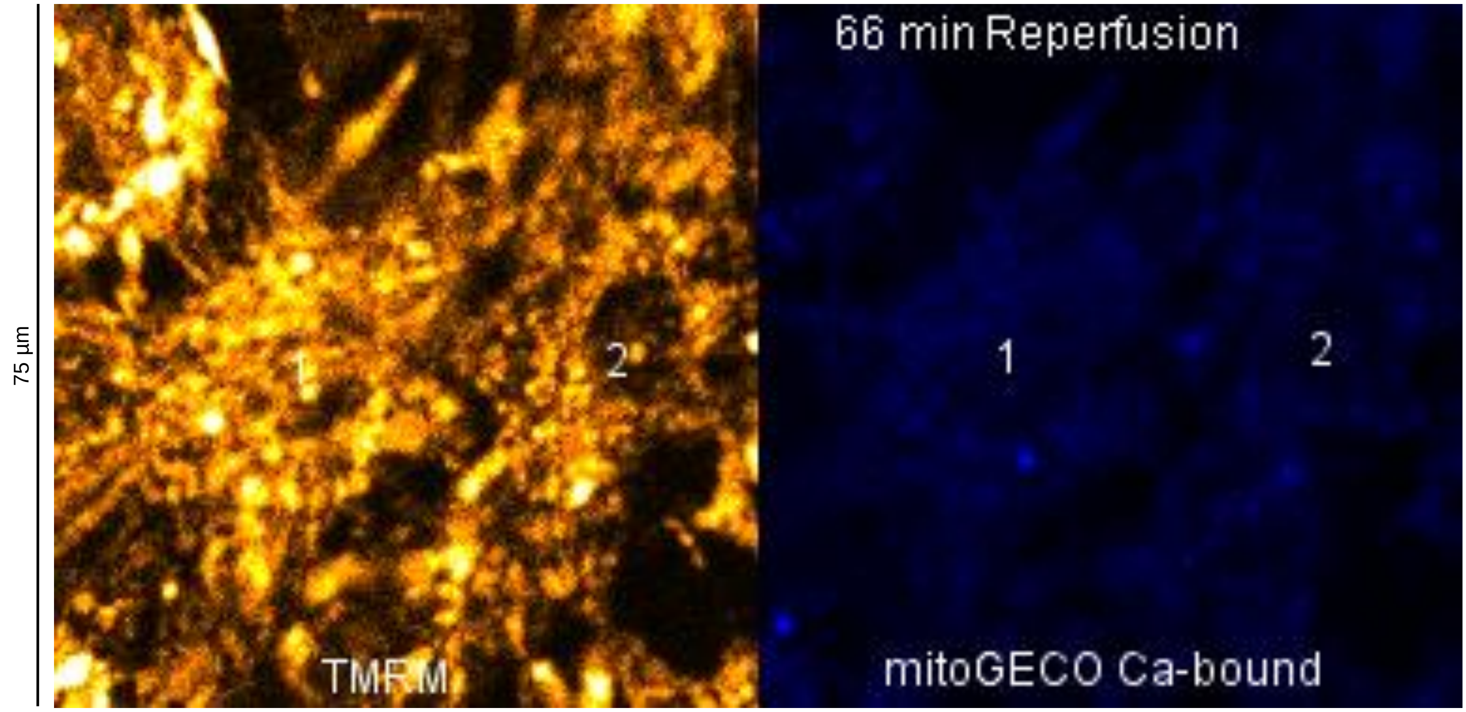

Video S3. ΔΨ Instability vs. mitochondrial [Ca^{2+}] during reperfusion. Video shows combined confocal images of 75x75 µm^2 area in the center of a monolayer transfected with mitoGECO and loaded with TMRM during reperfusion after 1 hour of ischemia; time resolution: 30 sec. Orange shows the TMRM signal, and blue is the Ca^{2+}-bound emission (475 nm) of mitoGECO. Mitochondria in cell 1 show an example of the behavior seen in Figures 6A and 6B; mitochondria in cell 2 show an example of the behavior seen in Figures 6C and 6D.

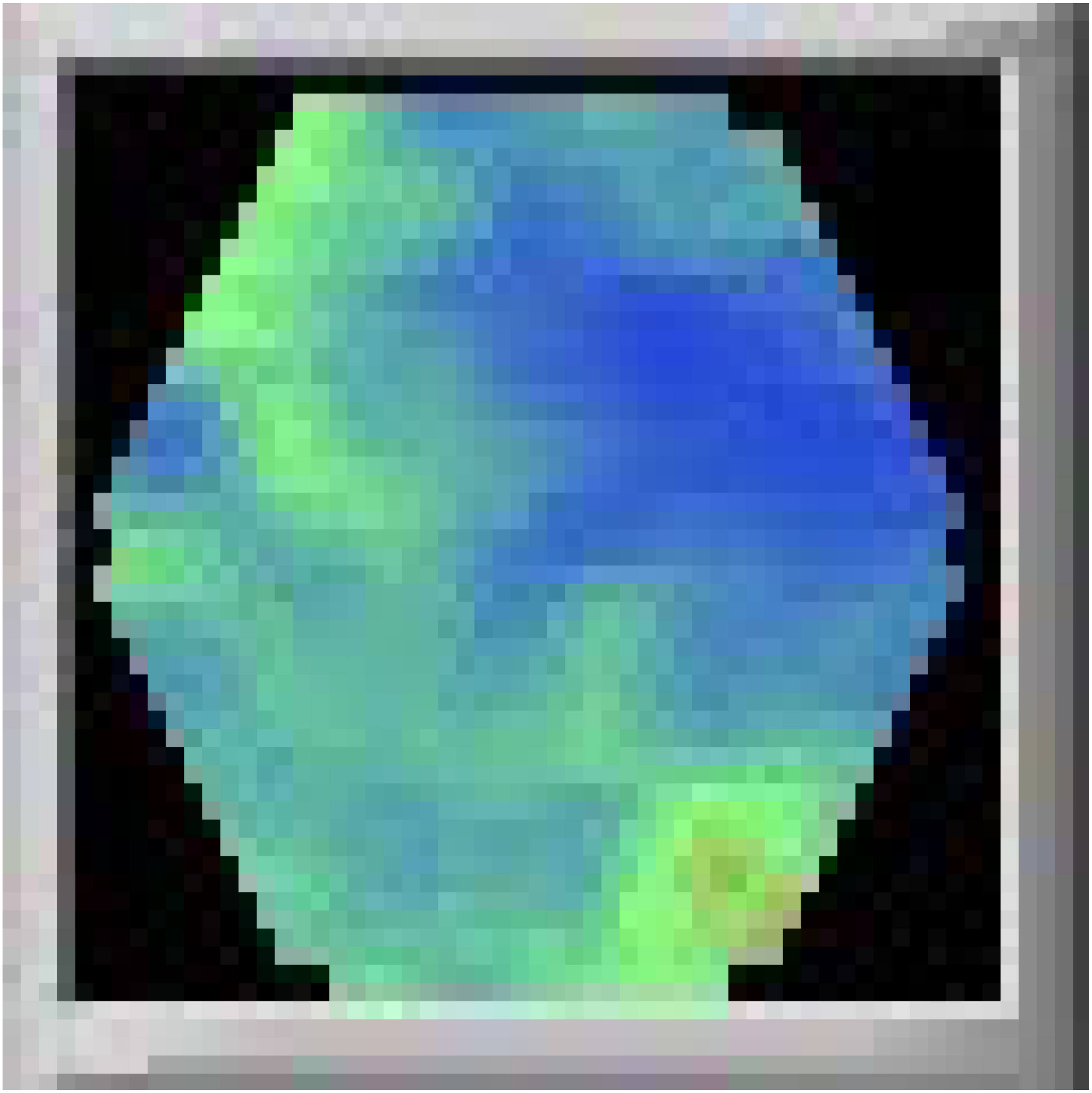

Video S4. Propagation of voltage waves through an NRVM monolayer preincubated with and in the presence of GSH-MEE. Voltage pulses are paced at 1Hz at 3 volts using bipolar point electrodes at the edge of the monolayer. A change from blue to red indicates depolarization of the sarcolemmal membrane.

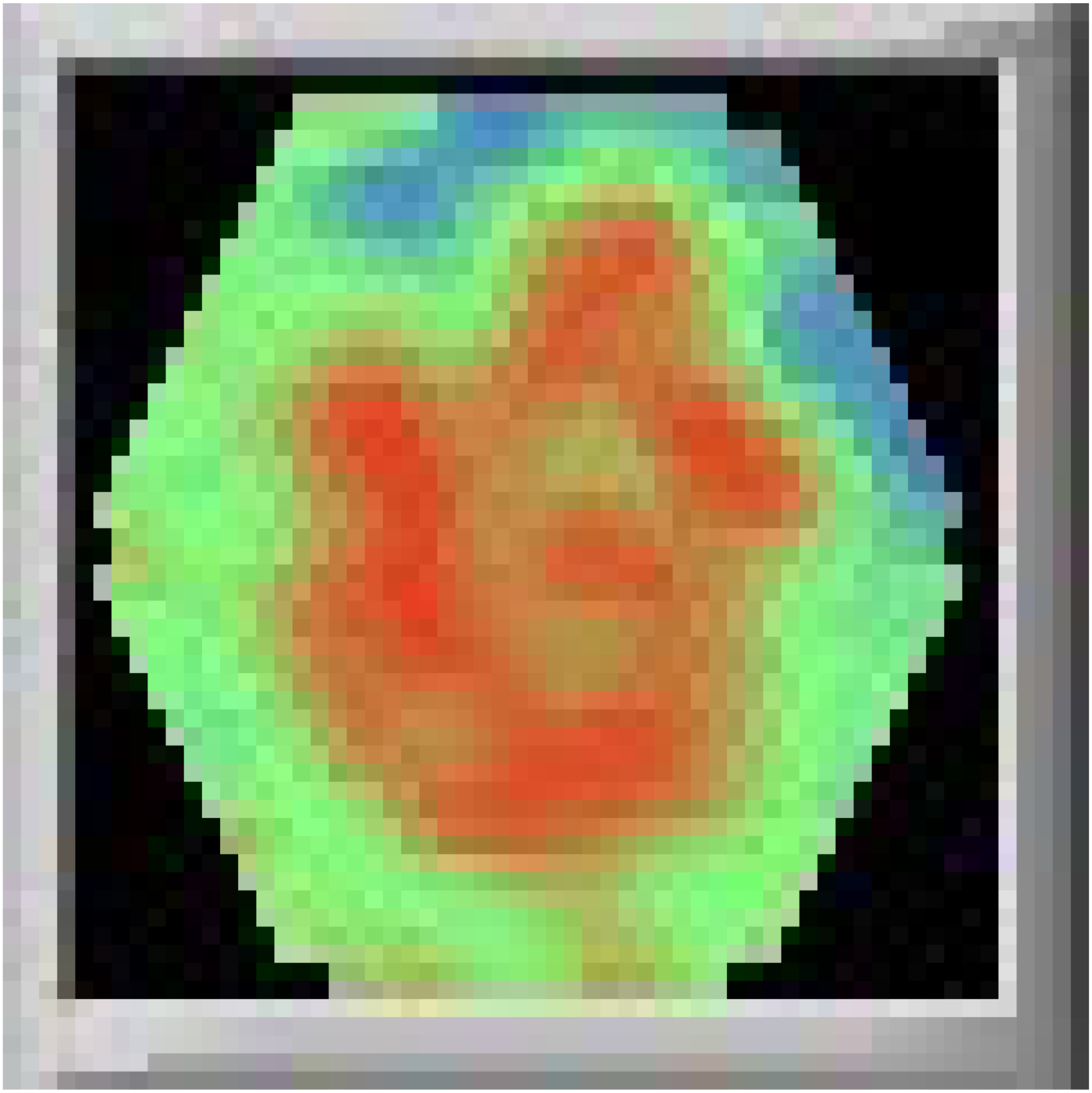

Video S5. Propagation of voltage waves around the ischemic area of an NRVM monolayer preincubated with and in the presence of GSH-MEE. Ischemia has been induced using a coverslip placed on the center of the monolayer. Voltage pulses go around the ischemic area, which is inexcitable. The apparent activity in the center (ischemic region) is noise augmented during video processing and does not represent action potentials. The monolayer is paced at 1Hz at 3 volts using bipolar point electrodes at the edge of the monolayer. A change from blue to red indicates depolarization of the sarcolemmal membrane.

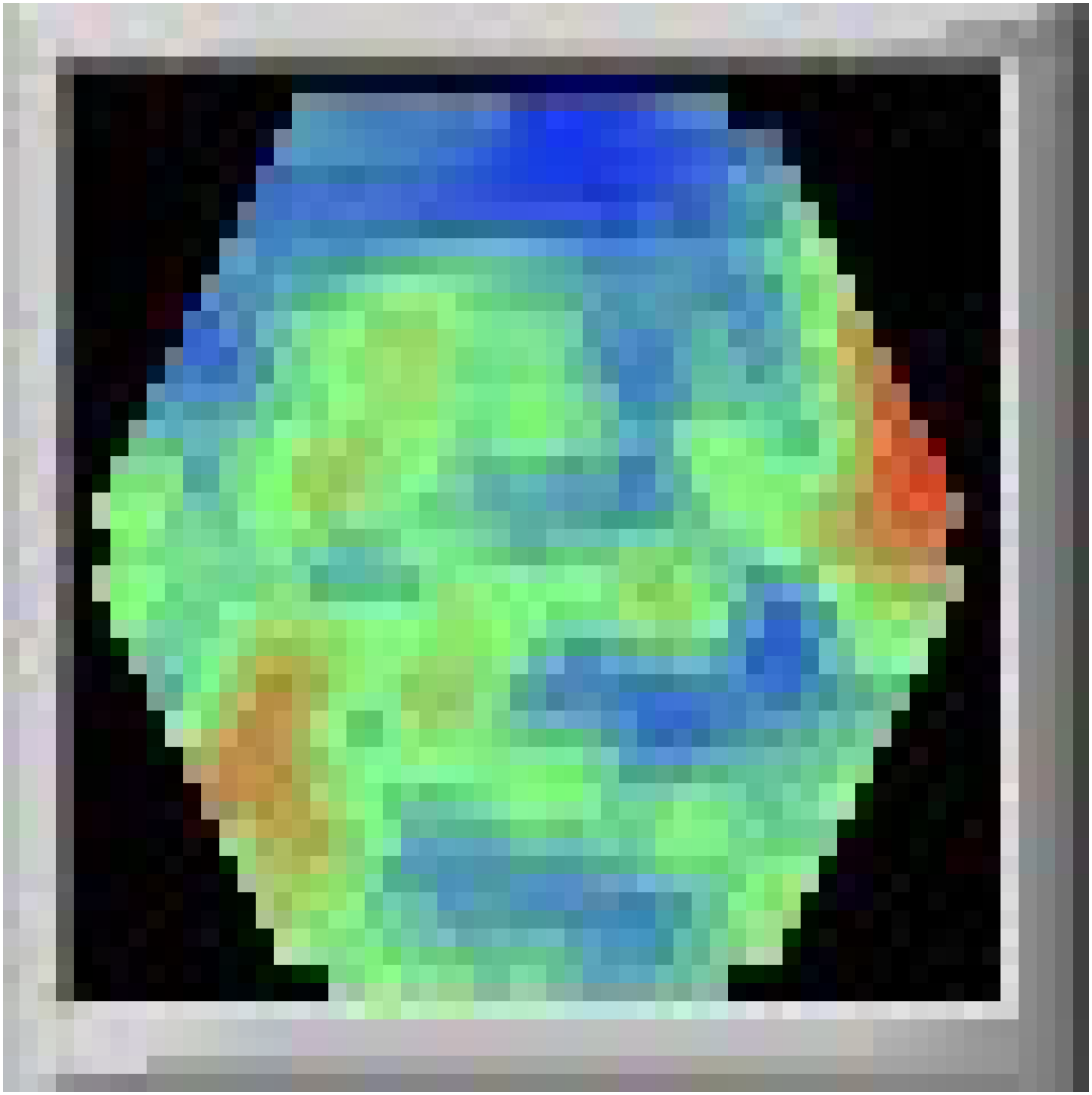

Video S6. Formation of wavelets and reentry upon reperfusion of an NRVM monolayer preincubated with and in the presence of GSH-MEE. The monolayer is paced at 1Hz at 3 volts using bipolar point electrodes at the edge of the monolayer. A change from blue to red indicates depolarization of the sarcolemmal membrane.
